# Astaxanthin Alleviates Hepatic Lipid Metabolic Dysregulation Induced by Microcystin-LR

**DOI:** 10.3390/toxins16090401

**Published:** 2024-09-18

**Authors:** Qinmei Tan, Hanyu Chu, Jia Wei, Sisi Yan, Xiaoya Sun, Jiangping Wang, Lemei Zhu, Fei Yang

**Affiliations:** 1 Hunan Province Key Laboratory of Typical Environmental Pollution and Health Hazards, School of Public Health, Hengyang Medical School, University of South China, Hengyang 421001, China; tqm2022@stu.usc.edu.cn (Q.T.); 2022000125@usc.edu.cn (S.Y.); sunxy089@163.com (X.S.); 2 Hengyang Maternal and Child Health Hospital, Hengyang 421001, China; hanyuchu@126.com; 3 Xiangya School of Public Health, Central South University, Changsha 410078, China; wjcindy@csu.edu.cn; 4 Hunan Engineering Research Center of Livestock and Poultry Health Care, Colleges of Veterinary Medicine, Hunan Agricultural University, Changsha 410128, China; wangjp9606@163.com; 5 School of Public Health, Changsha Medical University, Changsha 410219, China; zhulemei1228@163.com; 6 Affiliated Nanhua Hospital University of South China, Hengyang 421000, China

**Keywords:** astaxanthin, microcystin-LR, liver damage, lipid metabolism

## Abstract

Microcystin-LR (MC-LR), frequently generated by cyanobacteria, has been demonstrated to raise the likelihood of liver disease. Few previous studies have explored the potential antagonist against MC-LR. Astaxanthin (ASX) has been shown to possess various beneficial effects in regulating lipid metabolism in the liver. However, whether ASX could alleviate MC-LR-induced hepatic lipid metabolic dysregulation is as yet unclear. In this work, the important roles and mechanisms of ASX in countering MC-LR-induced liver damage and lipid metabolic dysregulation were explored for the first time. The findings revealed that ASX not only prevented weight loss but also enhanced liver health after MC-LR exposure. Moreover, ASX effectively decreased triglyceride, total cholesterol, aspartate transaminase, and alanine aminotransferase contents in mice that were elevated by MC-LR. Histological observation showed that ASX significantly alleviated lipid accumulation and inflammation induced by MC-LR. Mechanically, ASX could significantly diminish the expression of genes responsible for lipid generation (*Srebp-1c*, *Fasn*, *Cd36*, *Scd1*, *Dgat1*, and *Pparg*), which probably reduced lipid accumulation induced by MC-LR. Analogously, MC-LR increased intracellular lipid deposition in THLE-3 cells, while ASX decreased these symptoms by down-regulating the expression of key genes in the lipid synthesis pathway. Our results implied that ASX played a crucial part in lipid synthesis and effectively alleviated MC-LR-induced lipid metabolism dysregulation. ASX might be developed as a novel protectant against hepatic impairment and lipid metabolic dysregulation associated with MC-LR. This study offers new insights for further management of MC-LR-related metabolic diseases.

## 1. Introduction

The recurrent emergence of harmful cyanobacterial blooms (CyanoHABs) threatens ecological safety and public health. Microcystins (MCs), the main type of cyanobacterial toxins, are frequently found in freshwater bodies, with a complex array of over 310 variants [1,2]. In particular, microcystin-LR (MC-LR) is a standout congener for its ubiquity and toxicity, within the bounds of 1–10 micrograms per liter in natural water [3,4]. According to the International Agency for Research on Cancer (IARC), the carcinogenic risk of MC-LR is categorized under Group 2B [5,6]. Moreover, MC-LR is mainly taken up by eukaryotic cells through organic anion transporting polypeptides (OATPs) and interferes with the function of serine/threonine protein phosphatases (PP1 and PP2A), which contributes to multi-organ and multi-system toxicity, including hepatotoxicity, nephrotoxicity, and reproductive organ toxicity [7,8,9,10,11,12].

Recently, the influence of MC-LR on liver damage has attracted widespread attention. Evidence from both population-level and laboratory studies has suggested that MC-LR enhanced liver toxicity [13,14,15,16,17,18]. On the one hand, MC-LR might raise the chances of liver dysfunction occurring. For example, the findings from a survey in southwest China provide evidence that MC-LR may contribute to a higher chance of liver injury [13]. Moreover, based on a small population of fishers living near Taihu Lake (where cyanobacterial blooms are prevalent), Zhao et al. [14] documented a positive relationship between the presence of MCs and serum biochemical indices related to hepatic steatosis. On the other hand, ex vivo and in vivo experiments highlight the ability of MC-LR to cause hepatic lipid metabolic dysregulation related to oxidative stress, inflammation, apoptosis, and DNA damage [13,14,16,17,18,19]. Moreover, MC-LR markedly affects the expression of proteins associated with lipid metabolism, which, in turn, exacerbates the development of metabolic associated fatty liver disease (MAFLD) [13,14]. Our previous study also demonstrated that persistent contact with MC-LR resulted in liver tissue inflammation alongside lipid metabolic imbalances in mice, and the MAFLD symptoms might be aggravated when subjected to a high-fat diet [20,21]. However, the impacts of subacute MC-LR exposure on hepatic lipid metabolism have not yet been fully understood.

Due to the irreversible and severe damage to the liver caused by MC-LR, it is important to develop effective prophylaxis and therapy. However, few studies have been conducted on exploring potential chemoprotectants or antidotes against MC-LR. Previous studies suggested that *Lactobacillus fermentum*, oral cholestyramine, hawthorn fruit extract, and vitamin C could alleviate the toxic effects induced by MC-LR [22,23,24,25]. Astaxanthin (ASX) is a naturally lutein-like carotenoid that is produced by various organisms, especially marine organisms and microorganisms [26]. ASX exhibits a wide range of beneficial effects, making it a promising candidate for applications in health [27,28,29,30,31]. Supplementation of ASX not only showed no indications of toxicity or poisoning but also exhibited a beneficial pharmacological effect. Recent studies suggest an unambiguous association between ASX and the improvement of MAFLD, which indicates the potential of ASX in regulating liver lipid metabolism [27,28,29]. Moreover, ASX was demonstrated to improve liver damage induced by various environmental pollutants, including acetaminophen, carbon tetrachloride, and lipopolysaccharide [32,33,34]. Nevertheless, the precise impacts and mechanisms of ASX on hepatic lipid metabolic dysregulation triggered by MC-LR are poorly understood.

In this research, we innovatively explore the beneficial effects and underlying mechanisms of ASX on liver metabolism affected by MC-LR. Firstly, we evaluated the disruptions of lipid metabolism affected by MC-LR in mice, and subsequently assessed whether ASX could alleviate these symptoms. Furthermore, we investigated the critical mechanisms of ASX in regulating liver lipid metabolic dysregulation caused by MC-LR. Additionally, in vitro experiments were performed to further demonstrate our findings. We systematically uncovered the functional contributions of ASX in mitigating MC-LR-induced liver lipid metabolic disorders. This study provided novel perspectives on promising treatments for MC-LR-related metabolic diseases.

## 2. Results

### 2.1. Characteristics of Mice

**Figure 1:**
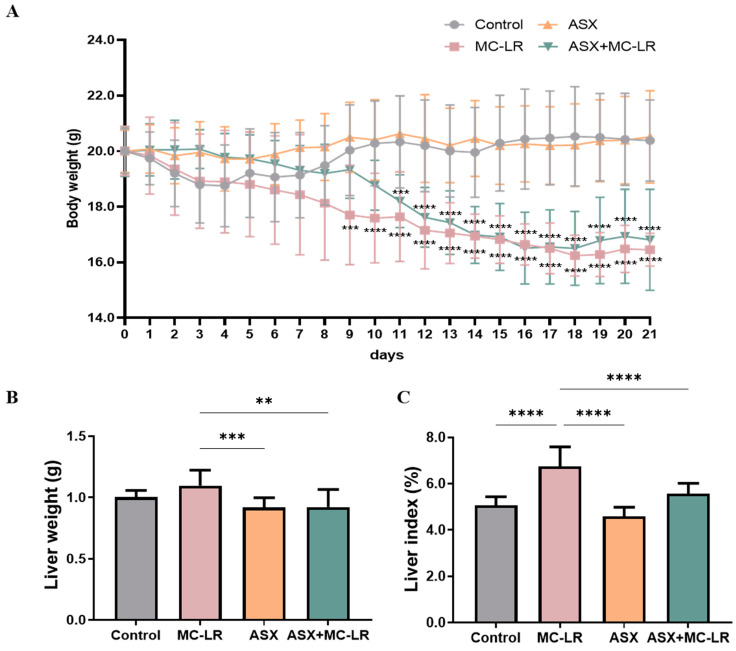
General features of mice in different groups. (**A**) Alterations of body weight. (**B**) Liver weight. (**C**) Liver index. Mean ± SD, ** *p* < 0.01, *** *p* < 0.001, **** *p* < 0.0001. N = 15, 10, 15, and 11 for the Control, MC-LR, ASX, and ASX+ MC-LR groups, respectively.

Figure 1A illustrates that body weights of mice in the control and ASX groups increased from the 7th day and then stabilized by the 15th day. Moreover, throughout the treatment period, no noteworthy divergences in the body weights were recorded in the comparison of the ASX group to the control group. By contrast, the body weights of mice within the MC-LR and the ASX + MC-LR groups gradually decreased. Notably, the MC-LR group showed a pronounced decrease in body weights compared to the control group starting from day 9, while the body weights within the ASX + MC-LR group mirrored this decrease from day 11. After 21 days, the liver weight and liver index of the MC-LR group were higher than those of the control group. However, the liver weight and liver index in the mice of ASX group remained equivalent to that of the control group. Furthermore, the ASX and the ASX + MC-LR groups had markedly lower liver weight and liver index than the MC-LR-only group (Figure 1B,C). The findings suggested that ASX not only had no adverse effect on body health but also showed the potential to delay the MC-LR-induced weight loss and improve liver damage.

### 2.2. Alteration of Biochemical Indices

**Figure 2:**
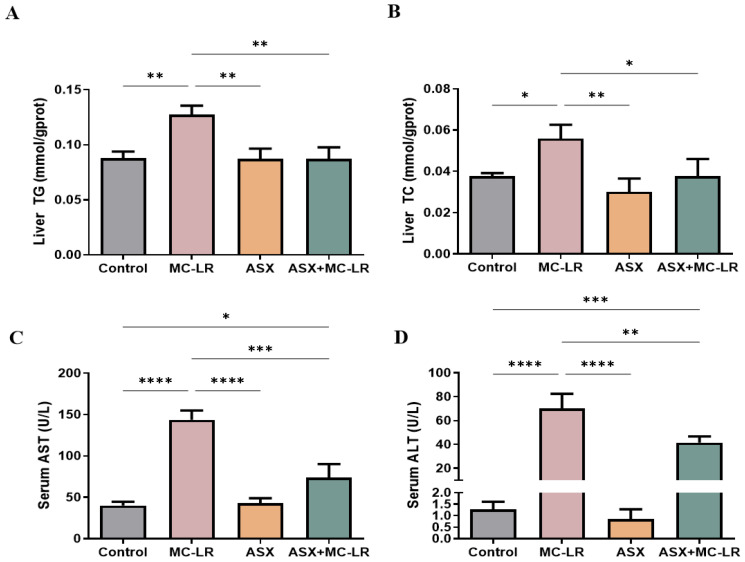
Liver and serum biochemical indices of mice in different groups. (**A**) The content of TG in liver. (**B**) The content of TC in liver. (**C**) The activity of AST in the serum sample. (**D**) The activity of ALT in the serum sample. Mean ± SD, * *p* < 0.05, ** *p* < 0.01, *** *p* < 0.001, **** *p* < 0.0001.

The contents of biochemical indices related to lipid metabolism and liver function were measured, including triglyceride (TG), total cholesterol (TC), aspartate transaminase (AST), and alanine aminotransferase (ALT) (Figure 2). The liver TG and TC levels and serum AST and ALT activities were significantly higher in the MC-LR group than in the control group. In contrast, a considerable decline in these liver and serum biochemical indices was recorded in both the ASX and ASX + MC-LR groups. Thus, MC-LR substantially impaired liver function and increased fat storage, while ASX could effectively reduce the symptoms triggered by MC-LR.

### 2.3. Histological Evaluation of Liver

**Figure 3:**
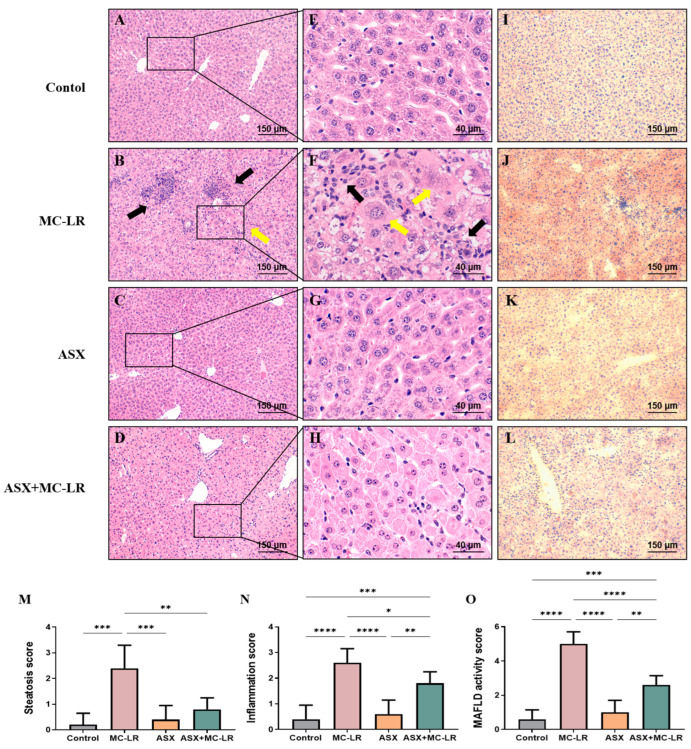
Histopathological changes in the livers of mice. (**A**–**D**) H&E staining of representative images of liver sections with 100× magnification (scale bar = 150 µm). (**E**–**H**) H&E staining of representative images of liver sections with 400× magnification (scale bar = 40 µm). (**I**–**L**) Oil Red O staining of representative images of liver sections (100× magnification, scale bar = 150 µm). Inflammatory cell clusters are indicated by black arrows, and enlarged liver cells are indicated by yellow arrows. (**M**) Steatosis score. (**N**) Inflammation score. (**O**) MAFLD activity score. Mean ± SD, * *p* < 0.05, ** *p* < 0.01, *** *p* < 0.001, **** *p* < 0.0001.

Hematoxylin and eosin (H&E) and Oil Red O staining of liver sections are shown in Figure 3. There was no pathological alteration observed in the control group and the ASX group. H&E staining suggested that the hepatic lobule structure was clear and the hepatocytes were organized in a radial configuration around the central vein in the control group (Figure 3A,E) and the ASX group (Figure 3C,G). In contrast, the lobules of liver in the MC-LR group (Figure 3B,F) appeared disordered, with enlarged hepatocytes, accompanied by inflammatory cell infiltration and hepatocellular ballooning. Notably, treatment with ASX seemed to alleviate the inflammation induced by MC-LR (Figure 3D,H). Moreover, Oil Red O staining indicated the absence of lipid droplets in both the control and ASX groups (Figure 3I,K). The red lipid droplets and fat vacuoles in the hepatocytes were noted within the MC-LR group (Figure 3J). However, the degree of lipid accumulation was significantly reduced in the ASX + MC-LR group (Figure 3L). Moreover, hepatic steatosis, inflammation, and MAFLD activity scores were all elevated following MC-LR exposure. However, the administration of ASX was found to reduce the elevated scores induced by MC-LR exposure (Figure 3M–O). Therefore, MC-LR could induce a certain extent of inflammation and lipid accumulation in the liver, while ASX had a great ability to alleviate these phenotypic manifestations.

### 2.4. Expression of Crucial Genes Involved in Liver Lipid Metabolism

**Figure 4:**
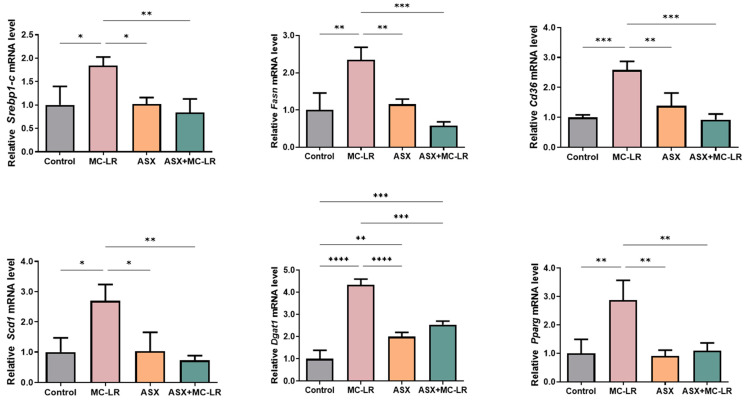
The expression levels of crucial genes related to liver lipid metabolism in mice. Mean ± SD, * *p* < 0.05, ** *p* < 0.01, *** *p* < 0.001, **** *p* < 0.0001.

To further illustrate the disturbance of liver lipid metabolism triggered by MC-LR and elucidate the molecular mechanisms of ASX in alleviating lipid accumulation, we uncovered the manifestation of genes governing liver lipid metabolism using qRT-PCR analysis. The MC-LR group showed a notable elevation in the relative mRNA levels of several genes responsible for lipid synthesis, including sterol regulatory element-binding protein-1c (*Srebp-1c*), fatty acid synthase (*Fasn*), CD36 molecule (*Cd36*), stearoyl-Coenzyme A desaturase 1 (*Scd1*), diacylglycerol O-acyltransferase 1 (*Dgat1*), and peroxisome proliferator-activated receptor gamma (*Pparg*), when compared to the control group (Figure 4). Notably, except for *Dgat1*, the mRNA levels of these genes in the ASX group and the control groups showed no statistical difference. Moreover, the relative mRNA levels of *Srebp-1c*, *Fasn*, *Cd36*, *Scd1*, *Dgat1*, and *Pparg* were profoundly higher in the MC-LR group than those in the control group. However, the relative mRNA levels of these genes were significantly lower in both the ASX group and the ASX + MC-LR group than those in the MC-LR group. These findings suggested that the exposure of MC-LR may contribute to liver lipid metabolic dysregulation. More importantly, ASX probably alleviated lipid accumulation induced by MC-LR by inhibiting the expression of lipid synthesis-related genes.

### 2.5. ASX Alleviated MC-LR-Induced Lipid Accumulation in THLE-3 Cells

**Figure 5:**
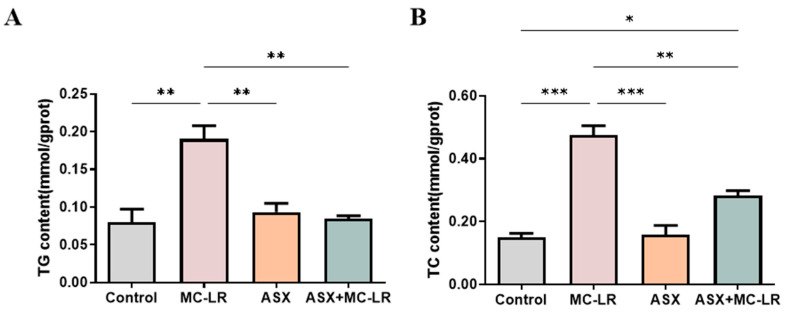
Alterations of the biochemical indicators in THLE-3 cells. (**A**) The content of intracellular TG. (**B**) The content of intracellular TC. Mean ± SD, * *p* < 0.05, ** *p* < 0.01, *** *p* < 0.001.

We verified the beneficial functions of ASX on MC-LR-induced liver lipid accumulation (Figure 5). Consistent with the animal experiments, the contents of biochemical indexes related to lipid metabolism were nearly the same in the ASX and control groups. Nevertheless, a significant surplus of intracellular TG and TC was noted in the MC-LR group, outstripping the levels found in the control group. Moreover, the intracellular TG and TC contents in both the ASX group and ASX + MC-LR group remarkably decreased, as opposed to the MC-LR group. Hence, MC-LR might promote lipid accumulation in THLE-3 cells, and ASX might lower the lipid accumulation induced by MC-LR.

### 2.6. ASX Regulated MC-LR-Induced Lipid Metabolic Dysregulation in THLE-3 Cells

**Figure 6:**
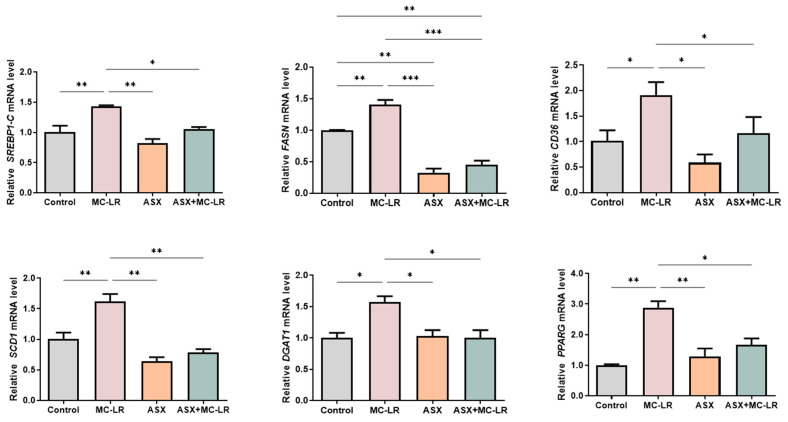
The expression levels of crucial genes associated with lipid metabolism in THLE-3 cells. Mean  ±  SD, * *p* < 0.05, ** *p* < 0.01, *** *p* < 0.001.

In contrast to the control group, a significant up-regulation of lipid synthesis-related genes (*SREBP-1C*, *CD36*, *SCD1*, *DGAT1*, and *PPARG*) was noted in the MC-LR group (Figure 6). Moreover, the expression of these genes (except for *FASN*) demonstrated no obvious change when comparing the ASX group to the control group. In addition, when pitted against the MC-LR group, both the ASX group and the ASX + MC-LR group exhibited a marked reduction in the expression of these genes in THLE-3 cells. Similarly, cell experiments also supported that MC-LR could result in lipid metabolic dysregulation, and ASX might improve the phenotypes induced by MC-LR. In particular, a decrease in the expression of genes responsible for the lipid synthesis pathway might contribute to the mechanisms.

## 3. Discussion

CyanoHABs and cyanotoxins have emerged as prominent concerns worldwide [35,36]. MC-LR is the best-known and broadly investigated cyanotoxin with high hepatotoxicity [37]. To date, most of the studies are dedicated to understanding the poisonous consequences of MC-LR, but few studies have explored the strategies to mitigate the toxicity of MC-LR. ASX is a kind of natural compound with various beneficial effects, playing a crucial part in liver metabolic health. Herein, a combination of in vivo and in vitro approaches was performed to innovatively elucidate the beneficial functions and molecular mechanisms of ASX on hepatic lipid metabolic dysregulation triggered by MC-LR. We demonstrated that subacute MC-LR exposure contributed to hepatic lipid metabolic dysregulation. ASX had a considerable potential to alleviate hepatic lipid metabolic dysregulation caused by MC-LR. Gene expression within the lipid synthesis pathway was probably responsible for the process. The results offered new insights for the management of MC-LR-related metabolic diseases.

Weight loss and liver injury were observed in mice after subacute exposure to MC-LR in this study. Previous studies also indicated that high doses of MC-LR resulted in a continuous decrease in body weight, accompanied by liver damage [38,39,40]. Consistently, our previous study demonstrated that long-term exposure to MC-LR had no significant effects on the body or liver weight of mice [20]. The principal drivers of this discrepancy might be the route, concentration, and duration of MC-LR exposure. The specific mechanisms need to be further elucidated. Moreover, our results suggested that serum TG and TC were increased by MC-LR. TG and TC were important indicators for evaluating the condition of lipid metabolism in the liver [41,42]. Qin et al. [40] also indicated that a fortnight of MC-LR exposure (0, 5, 10, and 20 µg/kg/d) had higher LDL-c levels in mice. Moreover, an epidemiological study suggested that serum TG and TC levels increased in fishermen consuming water with MCs more than they did in the control subjects [43]. In addition, we examined and found an enhancement of AST and ALT activities in this work. AST and ALT were considered appropriate diagnostic biomarkers for liver injury [44]. Zhao et al. [14] also reported that MC-LR exposure might up-regulate the contents of AST and ALT in mice. Consistently, according to the H&E and Oil Red O staining of liver sections, we observed that MC-LR exposure markedly induced lipid droplets and inflammation in mice. Lipid accumulation and inflammation were both representative manifestations of hepatic steatosis [45,46]. Collectively, subacute exposure to MC-LR resulted in weight loss in mice, along with the presence of liver injuries, and a notable increase in hepatic steatosis alongside liver inflammation.

In this study, although the body weight of mice might not be changed by ASX, it significantly observed that ASX reduced the liver injury and lipid accumulation as a consequence of MC-LR. Recent studies also suggested that ASX did not affect the body weight or liver weight of mice, but alleviated MAFLD symptoms in mice [47,48]. Moreover, Wu et al. [30] indicated that ASX could simultaneously improve the abnormal changes in body weight and liver indexes in mice. Many species could produce ASX, including shrimp, algae, and bacteria. ASX was extensively used as the ingredients of pharmaceuticals, cosmetics, and food additives, owing to its great function as an antioxidant and safety to humans [26]. Numerous clinical studies indicated that ASX significantly improved the severity of hepatic steatosis and delayed the development of inflammation, along with alleviating lipid metabolism-related diseases, including metabolic syndrome [49], and diabetes mellitus [50]. Moreover, some preclinical studies revealed the potential of ASX in alleviating liver injury alongside lipid deposition resulting from a diet rich in fat, since a series of lipid indicators like TG, TC, ALT, AST, lipid peroxides, inflammatory cytokines, and other elements were significantly improved by ASX [47,51,52]. In addition to unhealthy diets, some environmental pollutants were reported to facilitate liver injury. For instance, it was demonstrated that ASX could improve liver damage caused by carbon tetrachloride induced in murine models [33]. Nevertheless, none of the studies have investigated the roles and mechanisms of ASX on MC-LR-induced liver injury. In this study, pathological observation and biochemical analysis manifested that ASX could mitigate liver damage and decrease the extent of lipid accumulation induced by MC-LR. This is the first study to emphasize the beneficial effects of ASX on the toxicity of MC-LR. Our results confirmed that ASX exerted considerable potential in alleviating hepatic steatosis and inflammatory symptoms caused by various environmental toxins.

In addition, to further validate the protective benefits of ASX against MC-LR, we uncovered the important roles and molecular pathways of ASX in alleviating liver damage and lipid accumulation caused by MC-LR. Aligning with the results of animal experiments, MC-LR was proven to trigger fat deposition within THLE-3 cells. ASX could decrease the intracellular TG and TC levels of THLE-3 cells. THLE-3 cells, normal human liver epithelial cells, possess a degree of representativeness and have been widely used as a cellular model for hepatotoxicity [53,54,55]. However, the harmful impact of MC-LR on THLE-3 cells and the potential of ASX against MC-LR toxicity in THLE-3 cells have not yet been investigated. Our results were consistent with earlier reports, which indicated that MC-LR could induce lipid accumulation in hepatocytes [56]. Notably, other in vitro studies have demonstrated that ASX could protect hepatocytes from the stress of free fatty acids (FFAs) by decreasing lipid load and TG content in L02 cells [29,30]. We first demonstrated that ASX might improve lipid accumulation induced by MC-LR in THLE-3 cells.

Importantly, in this study, the lipid metabolism-related genes were significantly influenced by MC-LR and ASX. The impact of MC-LR was confirmed through animal and cellular experiments, revealing an up-regulation of *Srebp-1c*, *Fasn*, *Cd36*, *Scd1*, *Dgat1*, and *Pparg*. Fortunately, ASX significantly suppressed the expression of lipid metabolism-related genes activated by MC-LR. Liver lipid metabolism is an important metabolic process in the body, which includes synthesis, oxidation, and decomposition, and involves numerous genes [21]. *Srebp-1c* serves as a crucial regulatory factor in lipid synthesis, which activates and regulates key genes like *Fasn, Cd36, Scd1*, and *Dgat1* in lipid metabolism [57,58,59,60]. *Fasn* is essential for regulating the biosynthesis of endogenous fatty acids. *Cd36*, as an exogenous fatty acid transport enzyme, can promote long-chain fatty acid capture and transport. In the desaturation pathway of fatty acids, *Scd1* is regarded as a vital rate-limiting enzyme in the process. *Dgat1* mainly undertakes the responsibility for the synthesis of triglycerides in the process of fat absorption and storage [61,62,63,64,65,66,67,68]. *Pparg* also influences the metabolic pathways responsible for fatty acids and cholesterol [69,70,71]. Therefore, these genes were crucial molecules for evaluating lipid metabolic function in the liver. Abnormal expression of these genes has been extensively termed as biomarkers for lipid metabolism disorder. It was reported that MC-LR might increase the expression of *Srebp-1c*, *Fasn*, *Acc*, *Scd1*, and *Cd36* genes [20,39,40]. Moreover, a previous study suggested that ASX could down-regulate the expression levels of *Srebp-1c*, *Cd36*, *Acc*, *Fasn*, *Scd1*, and *Pparg* genes in mice consuming a high-fat diet, resulting in reduced fatty acid synthesis and lower lipid deposition in the liver [30,31,48]. Moreover, cell experiments also indicated that ASX significantly reduced the expressions of *Cd36* and *Srebp-1c* genes in hepatocytes [29,30]. Therefore, we innovatively revealed that ASX might improve liver lipid accumulation caused by MC-LR via regulating these crucial genes. 

This research proved for the first time that ASX could alleviate liver damage and lipid metabolic dysfunction associated with MC-LR. We preliminarily highlighted the effects and possible mechanisms of ASX on improving liver lipid accumulation triggered by MC-LR and that the expression of lipid synthesis genes was responsible for this process. Our discoveries presented convincing evidence for the potential application of ASX in managing MC-LR-related liver disease. Nevertheless, more specific mechanisms by which ASX alleviates MC-LR-induced liver injury and lipid metabolic dysregulation, such as oxidative stress, will be investigated in the future. Moreover, treating mice with oral MC-LR may be more consistent with the routes of human exposure in the real world. Treating mice with ASX after the MC-LR exposure is beneficial to assess the therapeutic effect of ASX on hepatic lipid metabolic dysregulation induced by MC-LR. In addition, hepatic organoid models, preclinical studies using other animals, and clinical studies are encouraged to strengthen our claims and offer a deeper insight into the mechanisms. 

## 4. Conclusions

In conclusion, subacute MC-LR exposure could contribute to liver damage and lipid metabolism dysregulation. We first proved that ASX acted as the potent antagonist against liver injury and lipid metabolic abnormalities caused by MC-LR. Moreover, results suggested that ASX presented protective mechanisms against MC-LR by regulating genes involved in the lipid synthesis pathway. This study uncovered the beneficial functions of ASX against MC-LR, offering a novel direction for the development of protectants against liver injury and lipid metabolic dysregulation resulting from MC-LR.

## 5. Materials and Methods

### 5.1. Animals and Experimental Design

**Figure 7:**
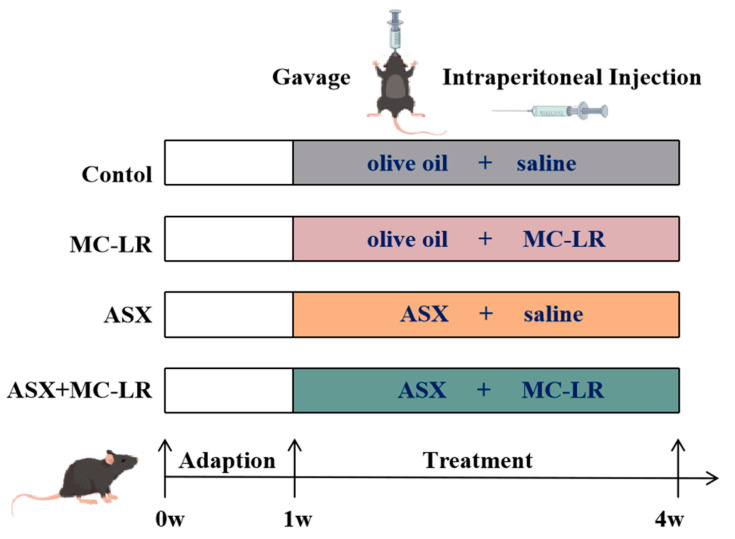
The procedure of mice experiments.

All the animal research was approved by the Ethics Research Committee of the University of South China (Approval number: USC202206XS09). Specific pathogen-free (SPF) male C57BL/6J mice (aged 6-8 weeks), purchased from Hunan SJA Laboratory Animal Co., Ltd., were housed in the Experimental Animal Centre of the University of South China. All the mice were kept in a SPF environment in controlled conditions (12 h light/dark cycle at 20–25 °C, humidity 70 ± 10%) with free access to food and water. The experimental design is illustrated in Figure 7. After 7 days of adaptive feeding, a total of 60 mice were methodically randomly allocated into 4 groups: the control group, the MC-LR group, the ASX group, and the ASX + MC-LR group. ASX was dissolved in olive oil and MC-LR was dissolved in saline. In the MC-LR group, mice were intraperitoneally (i.p.) injected with MC-LR (20 µg/kg/d bw) and oral gavage (i.g.) with olive oil. In the ASX group, mice were i.p. injected with saline and i.g. with ASX (50 mg/kg/d bw). In the ASX + MC-LR group, mice were i.p. injected with MC-LR (20 µg/kg/d bw) and i.g. with ASX (50 mg/kg/d bw). In the control group, mice were administrated with an equal amount of saline or olive oil. During the experiments, mice were fed with a chow diet and ad libitum. Body weight was recorded every day. After 21 days of treatment, a total of five mice died in MC-LR group and four mice died in MC-LR + ASX group probably because of high doses of MC-LR. The remaining mice were sacrificed, with subsequent collection of serum and liver tissues. The liver weight was measured, and the liver index was calculated using the formula: liver index = (liver weight/body weight) × 100%.

### 5.2. Histopathological Analysis

After fixing in 4% paraformaldehyde, liver samples were sent to Wuhan Sevier Biotechnology Co., Ltd. for H&E and Oil Red O staining. For H&E, the samples were dewaxed, stained with hematoxylin to show nuclei, then with eosin for cytoplasm and matrix, and mounted. For Oil Red O, fresh frozen sections were stained to reveal lipids, differentiated, lightly stained with hematoxylin, and mounted. Subsequently, the histopathologic phenotype of liver tissues was evaluated using an upright optical microscope (Motic, Xiamen, China). Liver pathology was scored according to an MAFLD activity scoring system [72]. The scores = steatosis (0–3) + inflammation (0–3) + ballooning (0–2) scores. Steatosis was scored 0 (<5%), 1 (5–33%), 2 (34–66%), and 3 (>66% of cells affected). Inflammation was graded 0 (no), 1 (<2), 2 (2–4), and 3 (>4 foci per 200× field). Ballooning was rated 0 (none), 1 (few), or 2 (many balloon cells).

### 5.3. Cell Culture

The THLE-3 cell line, a model of normal human liver epithelial cells, was donated by Professor Shu Weiqun at the Fourth Military Medical University. Cells were incubated in a humidified cell incubator (Thermo Fisher Scientific, Waltham, MA, USA) with 5% CO_2_ at 37 °C. THLE-3 cells in the logarithmic growth phase were categorized into four groups: the control group, the MC-LR group (20 µmol/L), the ASX group (50 µmol/L), and the ASX + MC-LR group (simultaneously exposed with 50 µmol/L ASX and 20 µmol/L MC-LR). MC-LR was dissolved in sterile PBS containing 10% dimethyl sulfoxide and ASX was dissolved in olive oil. After a 24-h treatment, cells were harvested for further experimental procedures.

### 5.4. Biochemical Indexes Determination

Commercial kits (Jiancheng, Nanjing, China) were used to determine TG (linear range, 0.3–11.4 mmol/L), TC (linear range, 0–19.39 mmol/L), AST (0–72.3 U/L), and ALT (0–72.3 U/L) concentrations in mice and cells following the manufacturer’s protocol. When the measured value exceeded the corresponding detection range, the sample was retested again after dilution.

### 5.5. Real-Time Quantitative PCR (RT-qPCR) Analysis

Total RNA was extracted using Trizol reagent, reverse transcribed using HiScript II Q RT SuperMix (Vazyme Biotech Co., Ltd., Nanjing, China), and qPCR was performed using ChamQ Universal SYBR qPCR Master Mix (Vazyme Biotech Co., Ltd., Nanjing, China). The primers and their sequences are shown in Table S1. The qPCR reactions were described in our previous report [73], using a three-step cycling protocol on a qTower3G instrument (Analytik Jena AG, Jena, Germany). The expression levels of the target genes were normalized relative to the expression of β-actin, which served as an endogenous control. All assays were performed in triplicate.

### 5.6. Statistical Analysis

Data were reported as the mean ± standard deviation (SD). Statistical analysis was performed on SPSS 26.0 (SPSS Inc., Chicago, IL, USA) and GraphPad Prism 9.0 software (GraphPad Software, San Diego, CA, USA). All data underwent one-way analysis of variance (ANOVA), with a post hoc Tukey test for multiple comparisons, setting statistical significance at *p* < 0.05.

## Data Availability

Data are contained within the article.

## Supplementary Materials

The following supporting information can be downloaded at: https://www.mdpi.com/article/10.3390/toxins16090401/s1, Table S1: The primer sequences required for the experiment.
