# Correction: Tan et al. Astaxanthin Alleviates Hepatic Lipid Metabolic Dysregulation Induced by Microcystin-LR. *Toxins* 2024, *16*, 401

**DOI:** 10.3390/toxins17020049

**Published:** 2025-01-22

**Authors:** Qinmei Tan, Hanyu Chu, Jia Wei, Sisi Yan, Xiaoya Sun, Jiangping Wang, Lemei Zhu, Fei Yang

**Affiliations:** 1 Hunan Province Key Laboratory of Typical Environmental Pollution and Health Hazards, School of Public Health, Hengyang Medical School, University of South China, Hengyang 421001, China; tqm2022@stu.usc.edu.cn (Q.T.);; 2 Hengyang Maternal and Child Health Hospital, Hengyang 421001, China; 3 Xiangya School of Public Health, Central South University, Changsha 410078, China; 4 Hunan Engineering Research Center of Livestock and Poultry Health Care, Colleges of Veterinary Medicine, Hunan Agricultural University, Changsha 410128, China; 5 School of Public Health, Changsha Medical University, Changsha 410219, China; 6 Affiliated Nanhua Hospital University of South China, Hengyang 421000, China

The authors wish to make the following corrections to this paper [1].

The primers of *H-FASN* in the Table S1 should be corrected as follows: “forward primer 5′-CATCGGCTCCACCAAGTC-3′ and reverse primer 5′-GCTATGGAAGTGCAGGTTGG-3′”.

Moreover, the authors added some detailed information for the experimental design as follows:

“N = 15, 10, 15, and 11 for the Control, MC-LR, ASX, and ASX+ MC-LR groups, respectively.” was added for the caption of Figure 1.

In Section 5.1. Animals and Experimental Design, “After 21 days of treatment, all mice were sacrificed, with subsequent collection of serum and liver tissues” changed to “After 21 days of treatment, a total of five mice died in MC-LR group and four mice died in MC-LR + ASX group probably because of high doses of MC-LR. The remaining mice were sacrificed, with subsequent collection of serum and liver tissues.”

“MC-LR was dissolved in sterile PBS containing 10% dimethyl sulfoxide and ASX was dissolved in olive oil” was added in Section 5.3. Cell Culture.

The errors were introduced during the writing process. The authors apologize for any inconvenience caused to the readers by these changes. The authors state these changes do not affect the scientific results and conclusions of the study. The original version of this manuscript has also been updated to reflect these corrections.

## References

[1] Tan Q., Chu H., Wei J., Yan S., Sun X., Wang J., Zhu L., Yang F. (2024). Astaxanthin Alleviates Hepatic Lipid Metabolic Dysregulation Induced by Microcystin-LR. Toxins.

