# Supplementary material for: Astaxanthin Alleviates Hepatic Lipid Metabolic Dysregulation Induced by Microcystin-LR
Source: Toxins (Basel). 2024 Sep 18;16(9):401. doi: 10.3390/toxins16090401 (PMC11435617; doi:10.3390/toxins16090401)
Supplement: Supplementary file 1 [file toxins-16-00401-s001.zip › toxins-3161186-supplementary.pdf]

Table S1. The primer sequences required for the experiment.

Primer name	Forward primer	Reverse primer
H-β-ACTIN	TGCGTGACATTAAGGAGAA	AAGGAAGGCTGGAAGAGT
H-SREBP-1C	GCTGTTGGTGCTCGTCTCCTTG	TGCTTGCGATGCCTCCAGAAGT
H-FASN	5′-CATCGGCTCCACCAAGTC-3′	5′-GCTATGGAAGTGCAGGTTGG-3′
H-CD36	TGGTGCTGTCCTGGCTGTGTT	ACTGTGTTGTCCTCAGCGTCCT
H-SCD1	AACCTGGCTTGCTGATGATGTG	AGGAGTGGTGGTAGTTGTGGAA
H-DGAT1	GCAACTACCGTGGCATCCTGAA	CCGCTGGGAAACACAGAATGGT
H-PPARG	GCTGAATCCAGAGTCCGCTGAC	GATCGCCCTCGCCTTTGCTTT
M-β-Actin	TCAAGATCATTGCTCCTCCTGAG	ACATCTGCTGGAAGGTGGACA
M-Srebp-1c	TGGAGACATCGCAAACAAG	GGTAGACAACAGCCGCATC
M-Fasn	GCCTCCGTGGACCTTATC	ACAGACACCTTCCCGTCA
M-Cd36	GGCAGGAGTGCTGGATTA	GAGGCGGGCATAGTATCA
M-Scd1	GGGAATAGTCAAGAGGCT	ACGAGGACGACAATACAA
M-Dgat1	GTGGGTTCCGTGTTTGC	CTCGGTAGGTCAGGTTGTCT
M-Pparg	TTCGCTGATGCACTGCCTAT	TGATCGCACTTTGGTATTCTTGG
